# Theoretical Studies of Intracellular Concentration of Micro-organisms’ Metabolites

**DOI:** 10.1038/s41598-017-08793-2

**Published:** 2017-08-22

**Authors:** Hai-Feng Yang, Xiao-Nan Zhang, Yan Li, Yong-Hong Zhang, Qin Xu, Dong-Qing Wei

**Affiliations:** 1 State Key Laboratory of Microbial Metabolism, and School of Life Sciences and Biotechnology, Shanghai Jiao Tong University, Shanghai, China; 2 Chongqing key Laboratory of Oral Diseases and Biomedical Sciences, Chongqing Municipal Key Laboratory of Oral Biomedical Engineering of Higher Education, and College of Stomatology, Chongqing Medical University, Chongqing, China; 3 Department of Chinese Traditional Medicine, Chongqing Medical University, Chongqing, China; 4 Medicine Engineering Research Center, and College of Pharmacy, Chongqing Medical University, Chongqing, China

## Abstract

With the rapid growth of micro-organism metabolic networks, acquiring the intracellular concentration of microorganisms’ metabolites accurately in large-batch is critical to the development of metabolic engineering and synthetic biology. Complementary to the experimental methods, computational methods were used as effective assessing tools for the studies of intracellular concentrations of metabolites. In this study, the dataset of 130 metabolites from *E. coli* and *S. cerevisiae* with available experimental concentrations were utilized to develop a SVM model of the negative logarithm of the concentration (-logC). In this statistic model, in addition to common descriptors of molecular properties, two special types of descriptors including metabolic network topologic descriptors and metabolic pathway descriptors were included. All 1997 descriptors were finally reduced into 14 by variable selections including genetic algorithm (GA). The model was evaluated through internal validations by 10-fold and leave-one-out (LOO) cross-validation, as well as external validations by predicting -logC values of the test set. The developed SVM model is robust and has a strong predictive potential (n = 91, m = 14, R^2^ = 0.744, RMSE = 0.730, Q^2^ = 0.57; R^2^_p_ = 0.59, RMSE_p_ = 0.702, Q^2^_p_ = 0.58). An effective tool could be provided by this analysis for the large-batch prediction of the intracellular concentrations of the micro-organisms’ metabolites.

## Introduction

Metabolic engineering has been widely used to raise the outputs of many significant small chemicals in different industrial and daily products, such as food, beverages, medicine and even enzymes. No matter these molecules are natural or synthesized, in order to increase the metabolic flux of the target molecules^1^, intracellular metabolism should be studied quantitatively. There are a large amount of experimental methods for identifying both metabolite concentrations and flux direction with the rapid development of high-throughput LC-MS technology and other analytical technologies. However, as the intracellular concentration of most metabolites is quite low (at the level of micro-mole), it is difficult to determine them by current precise analytical instruments^2^, especially in mass determination. It is still a huge challenge for experimental measurements in microenvironment, especially in microorganism cells. On the other hand, to enrich the intracellular metabolites is still quite difficult and may decrease the accuracy of measurement. Recently, there are an increasing number of computational and mathematical models for simulations of the cellular metabolism in metabolic engineering and synthetic biology^3–6^, which require the metabolites’ concentrations as basic parameters. In addition, metabolite concentration can also be used as a criterion for antibacterial discovery^7^, which further increases the demand for concentration determination.

However, few theoretical methods have been developed by far for prediction of the intracellular concentrations. As well known, absolute metabolite concentration is a bridge to a quantitative understanding of cellular metabolism, as concentrations affect both the free energies and rates of metabolic reactions^8, 9^. Commonly, based on the second law of thermodynamics, establishing a theoretical model requires quantitative information like metabolite concentration or metabolic fluxes, which are always interrelated. According to thermodynamics laws, a chemical reaction follows the Van’t Hoff equation as below (Equation 1),

1 $${\rm{\Delta }}G={\rm{\Delta }}{G}^{\theta }+{\rm{RT}}\,\mathrm{ln}\,{\rm{Q}}$$

where ΔG and ΔG^θ^ are the non-standard and standard Gibbs free energy change of the reaction, and Q is the reaction quotient, *i.e*., the ratio of the chemical activities of products and reactants within the compartment where the reaction is occurring^9^. This equation dictates that net flux occurs in the reaction direction with ΔG < 0. Thus, metabolic fluxes direction is fundamentally and directly affected by absolute metabolite concentration. According to the relationship between metabolite concentrations and flux directions, unknown flux directions can be predicted from metabolite concentrations, vice versa, unknown metabolite concentrations can be predicted from known flux directions. Kummel *et al*. established a network embedded thermodynamic (NET) method to predict intracellular metabolite concentration, but the NET method needs Gibbs free energy of the metabolic reaction as the prior condition, which greatly limits its application scope because of the difficulty in Gibbs free energy measurement^10^. Hamilton *et al*. developed a method named thermodynamics-based metabolic flux analysis (TMFA), which is the developed from the general flux balance analysis and thermodynamic constraints analysis^11^. The TMFA method can predict an approximate range of metabolite concentrations based on relatively few information, which is more suitable for qualitative analysis rather than accurately quantitative analysis.

At the same time, prediction of metabolites’ concentrations was also attempted by statistical methods. With the rapid development in microorganism metabolic network, the relationship between metabolic network and chemical reactions was explored after reconstructing metabolic network. Since most biological metabolites are small molecules and metabolic processes are basically chemical reactions, to some extent, the metabolic network organization has chemical basis, which was discussed by Zhu *et al*. with combination of bioinformatics and cheminformatics^8^. It is possible to develop theoretical methods for predicting the intracellular concentration. Bar-Even *et al*. found that the hydrophobicity and charge of metabolites has great influence on the intracellular concentration of metabolites, and initially established a rough linear model for metabolites concentration prediction based on physical and chemical properties for the first time^12^. In the following researches, Zhu *et al*. found that there is a certain correlation between biological phenotype and metabolic network topology, in addition to physical and chemical properties, based on which a support vector machine model was established to predict the intracellular metabolite concentrations^8^. However, while the squared cross-validation correlation coefficients (Q^2^) of this model reached 0.59 in the internal validation in *E. coli*, it had no external validation with independent test set.

In addition to molecular properties, it might be helpful to utilize information of metabolites’ biological functions in the concentration prediction, since the functions are generally affected by the biological characters like the intracellular concentrations. However, in the earlier studies^8, 10–12^, less biological information was employed to predict metabolite concentrations. In this paper, in addition to molecular descriptors of structural and physicochemical properties, topological parameters in organism specific metabolic network and a novel type of parameters describing the involvement of a metabolite in specific pathways were utilized to improve the predictive performance, with discussion on their biological meaning. All these variables were applied to variable selection using genetic algorithm (GA)^13–15^ and an additional optimization procedure. Incorporated with the support vector machine algorithm^16–20^, the variable set was iteratively optimized to build the final prediction model^21^ which has the best performance in internal validation. The final model was then applied to the external validation in the test set, which was randomly selected from a dataset containing the experimental concentrations of 130 metabolites from two kinds of microorganisms, 93 metabolites from *E. coli* ^9^ and 37 metabolites from *S. cerevisiae* ^22^.

## Results

### Variable selection and interpretation

#### Variable selection

After a preprocessing, the variable selection is performed on 1669 variables step by step, including three rounds of GA selection and a further optimization procedure. The results are evaluated by both 10-fold cross-validation and leave-one-out (LOO) validation, as shown in Table 1. In the first five rounds of selection, the Root Mean Square Error (RMSE) is decreasing and the correlation coefficient (Q^2^) between computational and experimental values is increasing. Until in the fifth round, when the number of selected variables m is lowered to 14, the best performances are obtained both in 10-fold cross-validation with RMSE = 0.741, Q^2^ = 0.55 and in LOO method with RMSE = 0.730, Q^2^ = 0.57. In the next three rounds, the performances of the models are not improved, but drop a little bit. And in the ninth round, the dramatic increase in RMSE to 0.853 and decrease in Q^2^ to 0.41 in 10-fold cross-validation, as well as to 0.828 and 0.45 in LOO respectively, convinced us to stop further attempts.

**Table 1:** The variable selection.

Round	Number of variables(m)	10-fold cross-validation		Leave-one-out(LOO)	
		RMSE	Q^2^	RMSE	Q^2^
Initial	1669	1.120	0	1.103	0.02
1	697	1.053	0.13	1.050	0.13
2	258	0.973	0.23	0.960	0.25
3	70	0.912	0.33	0.898	0.35
4	18	0.771	0.52	0.769	0.52
**5**	**14**	**0.741**	**0.55**	**0.730**	**0.57**
6	13	0.772	0.52	0.758	0.54
7	12	0.786	0.50	0.756	0.54
8	11	0.780	0.51	0.751	0.55
9	10	0.853	0.41	0.828	0.45

Generally the variables’ number is fewer in a model, the model quality is much better. However, taking into account the number of training set samples, 91 is much bigger than the number of variables, 14, finally 14 descriptors are picked out as the variable set for the final SVM model described below. Detailed information about the 14 variables is shown in Table 2.

**Table 2:** The selected 14 variables of the optimal variable set.

Name	Type	Description
Clustering-Coefficient	Topological parameter	Clustering coefficients of nodes
Degree	Topological parameter	Degree of nodes
BCUT_SLOGP_2	Molecular descriptor	LogP BCUT (2/3)
BCUT_SMR_3	Molecular descriptor	Molar refractivity BCUT (3/3)
GCUT_PEOE_1	Molecular descriptor	PEOE charge GCUT (1/3)
SlogP_VSA9	Molecular descriptor	Bin 9 SlogP (0.40, 10]
PEOE_VSA + 0	Molecular descriptor	Total positive 0 vdw surface area
PEOE_VSA + 5	Molecular descriptor	Total positive 5 vdw surface area
Vsa_hyd	Molecular descriptor	VDW hydrophobe surface area
Opr_nring	Molecular descriptor	Oprea ring count
6mem_rings_molecules	Molecular descriptor	Number of 6 membered rings
RPCG	Molecular descriptor	Ratio of most positive charge on sum total positive charge (Relative positive charge)
ClogP	Molecular descriptor	Partition coefficient octanol/water
MPF descriptor	Metabolic pathway	Five Metabolic Pathways’ Features descriptor

#### Variable interpretation

In Table 2, there are three types of variables in the optimal variable set, including topological parameters of metabolic networks, molecular descriptors and metabolic pathway descriptors. It is obviously that molecular chemical descriptors are the main parts in the optimal variable set. In order to quantitatively measure the importance of each variable to the model, we calculate the correlation coefficients between each variables and –logC in Table 3, sorting by their absolute values. Because MPF is a categorical variable, here its correlation with –logC is calculated by Spearman Correlation Coefficient, while the other 13 variables are evaluated by Pearson Correlation Coefficient.

**Table 3:** Correlation coefficients between selected variables and-logC.

Variables	correlation coefficients
BCUT_SLOGP_2 MPF	0.446
	−0.437
Degree	−0.325
6mem rings Molecules	0.296
opr_nring	0.296
ClogP	0.267
GCUT_PEOE_1	0.235
Clustering Coefficient	−0.124
vsa_hyd	0.099
RPCG	−0.091
PEOE_VSA + 0	−0.075
PEOE_VSA + 5	−0.062
SlogP_VSA9	−0.035
BCUT_SMR_3	−0.024

As we can see from Tables 2 and 3, chemical character is still the most significant factor for the intracellular concentration of metabolites. The molecular descriptors in the optimal variable set are mainly related to four types of molecular physical-chemical properties: 1) molecular polarity, such as BCUT_SLOGP_2 and ClogP; 2) partial charge distribution, such as BCUT_SMR_3, GCUT_PEOE_1 and RPCG; 3) subdivided surface areas, such as SLOGP_VSA9, PEOE_VSA + 0, vsa_hyd; 4) geometric structure of molecules, such as opr_nring, 6men_rings_molecules. In these descriptors, 6mem_rings_molecules, RPCG and ClogP also appeared in Zhu’s model, while the other descriptors are also similar to those in the models of Zhu *et al*.^8^ or Bar-Even *et al*.^12^.

Topological parameter is another type of variable in the optimal variable set. Different with the Zhu’s variable set^8^, in addition to the parameter Degree, this optimal variable set contains a metabolic network topology variable named as Clustering-Coefficient, which represents the density of the network around the specific nodes. In graph theory, Clustering-Coefficient reflects the tendency of nodes to cluster together. If one node is connected with *n* other nodes, Clustering-Coefficient is the ratio of the number of edges between n nodes and the maximum number of potential maximum edges (${{\rm{C}}}_{n}^{2}$)^23^ (Equation 2).

2 $${\rm{Clustering}}\,{\rm{Coefficient}}=\,\frac{2\times {\rm{number}}\,{\rm{of}}\,{\rm{edges}}\,\mathrm{between}\,\,{\rm{n}}\,\,\mathrm{nodes}}{{\rm{n}}({\rm{n}}-1)}$$

**Figure 1:**
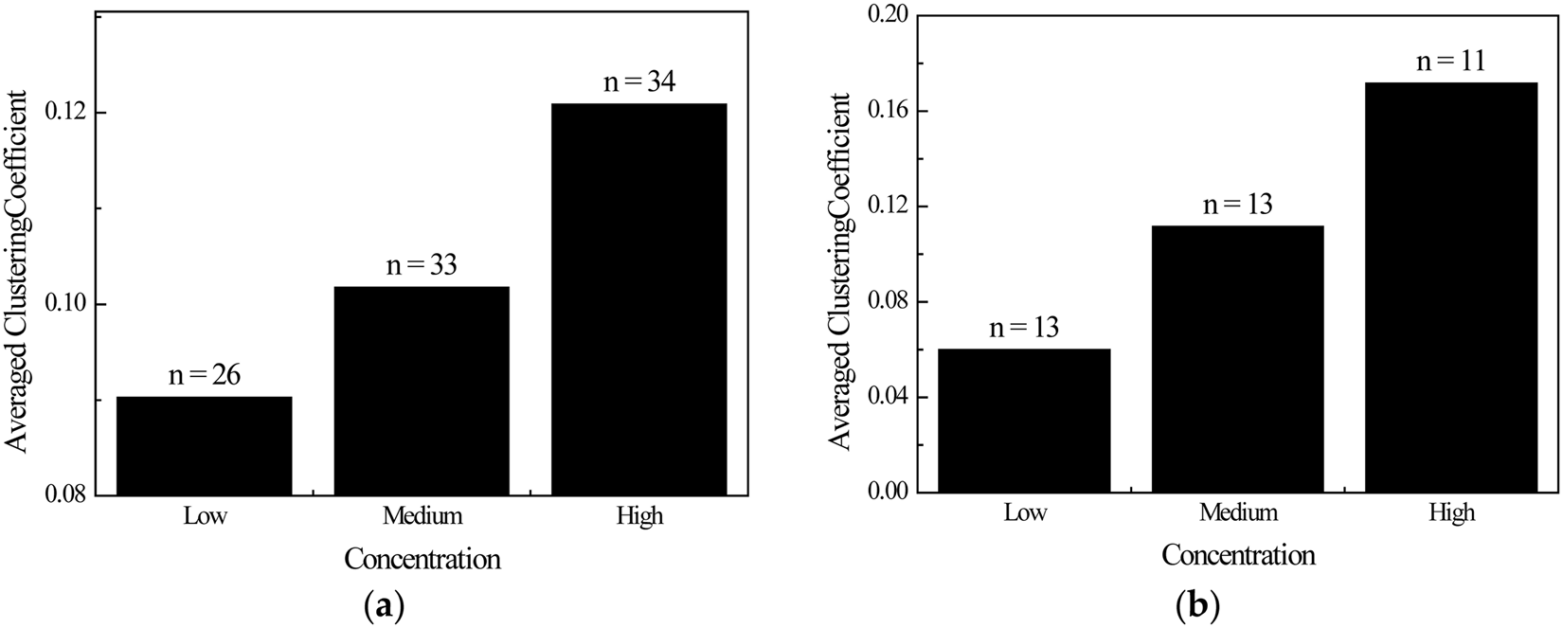
Correlation between metabolite concentration and Clustering-Coefficient in (**a**) *E. coli*; and (**b**) *S. cerevisiae*.

In metabolic biology, Clustering-Coefficient in metabolic network represents the concentration of metabolic reactions around the specific metabolite, which means that the more intensive the biochemical reactions around the metabolites, the higher concentrations of the metabolites are often required to ensure that they won’t be the bottleneck of the surrounding metabolic reactions. Correlations between metabolite concentration and Clustering-Coefficient in the dataset are shown in Fig. 1.

As in Fig. 1, the concentrations of the 93 metabolites of *E. coli* and the 37 metabolites of *S. cerevisiae* were roughly equally separated into three groups as Low, Medium, and High (26 concentrations in the range of 1.0 × 10^–7^–3.0 × 10^−5^ mol/L, 33 concentrations within 3.0 × 10^−5^–5.5 × 10^−4^ mol/L and 34 concentrations within 5.5 × 10^−4^–1.0 × 10^−1^ mol/L for *E. coli*, as well as 13 concentrations of 2.0 × 10^−5^–3.5 × 10^−4^ mol/L, 13 concentrations within 3.5 × 10^−4^–1.5 × 10^−3^ mol/L and 11 concentrations within 1.5 × 10^−3^−1.0 × 10^−1^ mol/L for *S. cerevisiae*, respectively). In both figures, the average metabolite concentrations are positively correlated with the average Clustering-Coefficient of the group of metabolites. Combining with the correlation coefficients in Table 3, it was indicated that Clustering-Coefficient may be a good variable to help improve the prediction model.

The third type of variable is metabolic pathway variable. As in Table 2, there is one selected variable named as Metabolite Pathways’ Feature descriptor (MPF descriptor), which describes the participation of a metabolite in five metabolic pathways in microbe metabolic network, that is, Map00625, Map00626, Map02020, Map03070 and Map04122. There are 14 metabolites involved in these pathways, with detailed information listed in Table S1 (see Supplementary information S1). In these five metabolic pathways, Map00625 represents the degradation pathway of chloralkane and chloroalkene, Map00626 represents the degradation pathway of naphthalene, Map02020 and Map04122 represent the pathways of signal transduction, and Map03070 represents the secretary pathway of bacteria. The five pathways selected are non-core metabolic pathways, that is, they are distinct from the core metabolic pathways that generally refer to the tricarboxylic acid cycle, such as glucose synthesis and decomposition.

**Figure 2:**
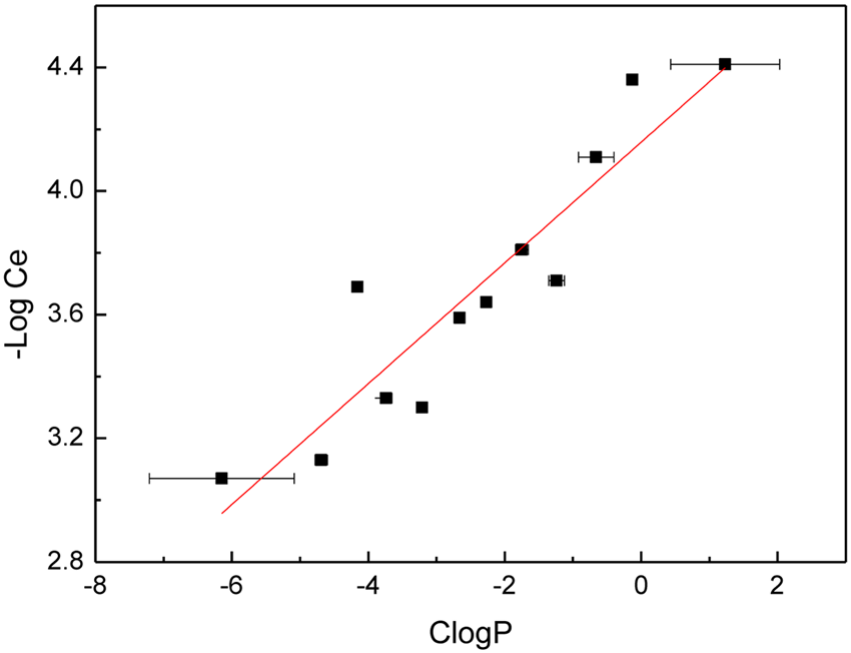
Correlations between concentration and CLogP of 130 metabolites (R^2^ = 0.838).

The reason why the pathway variables of these five non-core pathways are retained in the final model might be explained by the deviation of the concentrations and polarities of the 14 metabolites in these pathways from the average level. In microbe metabolic pathways, the concentration and the polarity of the metabolites are two key points. In intracellular micro-environment, the concentration and polarity of the metabolites are generally positively correlated^24^, although not strictly linear. This positive correlation was shown in Fig. 2. The 130 metabolites were first divided into 12 groups according to their values of CLogP with the bin width of 0.5, then the average values of CLogP and –logCe of the 12 groups were plotted, where CLogP is the metabolites’ partition coefficient in octanol/water directly correlated to their molecular polarity, while –logCe is the negative logarithm of their experimental intracellular concentrations. As shown in Fig. 2, the metabolite intracellular concentrations are positively correlated to CLogP, with R^2^ as high as 0.838, suggesting that metabolites more polar and thus more water-soluble may have higher intercellular concentrations. Therefore, the metabolite concentration could be predicted by chemical basis, at least partially.

In Table 4, the average CLogP of the 14 selected metabolites is −2.98, slightly lower than the average value of the 130 metabolites, −2.85, which indicates that the average polarity of the 14 metabolites is slightly higher than that of all 130 metabolites. On the other hand, in Table 4 the average -logC_e_ of the 14 metabolites in *E. coli* and *S. cerevisiae* are 2.77 and 2.32, respectively, much smaller than the overall average value as 3.57 of all the 130 metabolites in both organisms. It means that these 14 metabolites may have a lower polarity, but a higher concentration. This is a good example that the positive correlation between polarity and concentration of metabolites may vary in certain pathways of metabolites, such as the 14 metabolites in the five pathways of MPF descriptor. Therefore, this MPF descriptor can provide a contribution to complement the concentration prediction as an important variable.

**Table 4:** The deviation of the concentrations and polarities of the 14 metabolites in the five pathways included in the MPF descriptor.

Name	–log C_e_		CLogP
	*E. coli*	*S. cerevisiae*	
Glutamate	1.02	1.09	−2.69
ATP	2.02	2.47	−4.55
Aspartate	2.37	1.80	−2.41
Glutamine	2.42	1.09	−3.38
Citrate	2.71	2.83	−2.00
Malate	2.77	2.77	−1.52
Acetyl-CoA	3.22	NA	−3.54
Succinate	3.24	3.47	−0.53
Succinyl-CoA	3.63	NA	−3.94
Fumarate	3.94	2.78	−0.17
S-adenosyl-L-methionine	3.74	NA	−5.08
Alanine	2.59	1.61	−3.12
GTP	2.31	3.23	−5.53
D-Glucose 6-phosphate	NA	2.43	−3.28
Average of 14 above metabolites	**2.77**	**2.32**	−**2.98**
Average of 130 metabolites	**3.57**		−**2.85**

-log C_e_ is the negative logarithm of the corresponding concentration in *E. coli* and *S. cerevisiae*. NA means no data available.

### SVM regression model

The prediction model of metabolite concentration employing 14 descriptors was built by SVM regression^25^ based on 91 samples in training set, and then it was tested by the independent test set containing 39 samples. The confidence interval of 20 randomized trials is: R^2^ = 0.75 ± 0.02, RMSE = 0.746 ± 0.020, Q^2^ = 0.54 ± 0.05; R^2^_p_ = 0.56 ± 0.05, RMSE_p_ = 0.744 ± 0.041, Q^2^_p_ = 0.53 ± 0.05. The performance of the model is evaluated by randomly choosing one result as in Table 5. It is shown that with the sample size n = 91 and the feature size m = 14, the SVM regression model results in *Q* ^2^ = 0.55 and *RMSE* = 0.74 in the 10-fold cross-validation and *Q* ^2^ = 0.57, *RMSE* = 0.730 in the LOO cross-validation, which both suggest a good stability. Moreover, in the external validation by the independent test set, the predicted vs experimental –logC values have Q^2^_p_ = 0.58, R^2^_p_ = 0.586, RMSE = 0.702, which also proved the good reliability of this model. In order to further demonstrate the robustness of the model, the variance of prediction error in the LOO cross-validation and the independent test was calculated as 0.53 and 0.52, respectively. Compared with previous studies, our model was further testified by external validations and showed good reliability.

**Table 5:** Predictive performance among internal and external validation.

	Training set						Test set (n = 39)		
	Number of samples (n)	R^2^	10-fold		LOO		R^2^_p_	RMSE_p_	Q^2^_p_
			RMSE	Q^2^	RMSE	Q^2^			
Zhu’s model^8^	80	0.683			0.729	0.59			
Bar-Even^11^	60	0.43				0.43			
This model	91	0.744	0.741	0.55	0.730	0.57	0.586	0.702	0.58

In addition, 11 metabolites from *Bacillus subtilis* ^26^, which did not exist in our training set, were used to test the generalization ability in other micro-organisms, getting a good result of RMSE = 0.71. However, this data set comes from a different experimental protocol that may lead to some systematic error in the concentration prediction.

**Figure 3:**
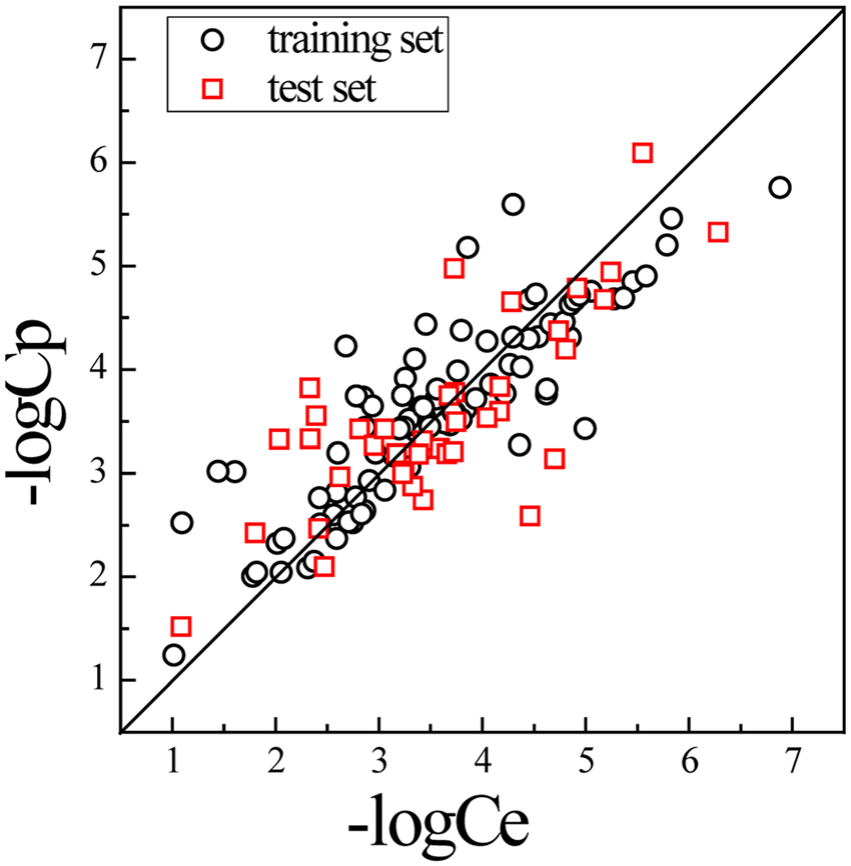
Plot of the -logC values predicted by the SVM model (-logCp) vs. those observed (-logCe).

The plot of the predicted vs. experimental –logC values is shown in Fig. 3, where both the sample of the training set as shown in black circles and those of the test set as shown in red squares are distributed near the diagonal, suggesting that the SVM regression model fits well and may estimate the intracellular metabolite concentrations with a good reliability.

The distribution of prediction errors is shown in Figure S1 (see Supplementary information S1). The prediction errors in both training set and test set obeys normal distribution approximately, which indicates that good performance of our model is not due to over-fitting.

### Application domain of the SVM model

**Figure 4:**
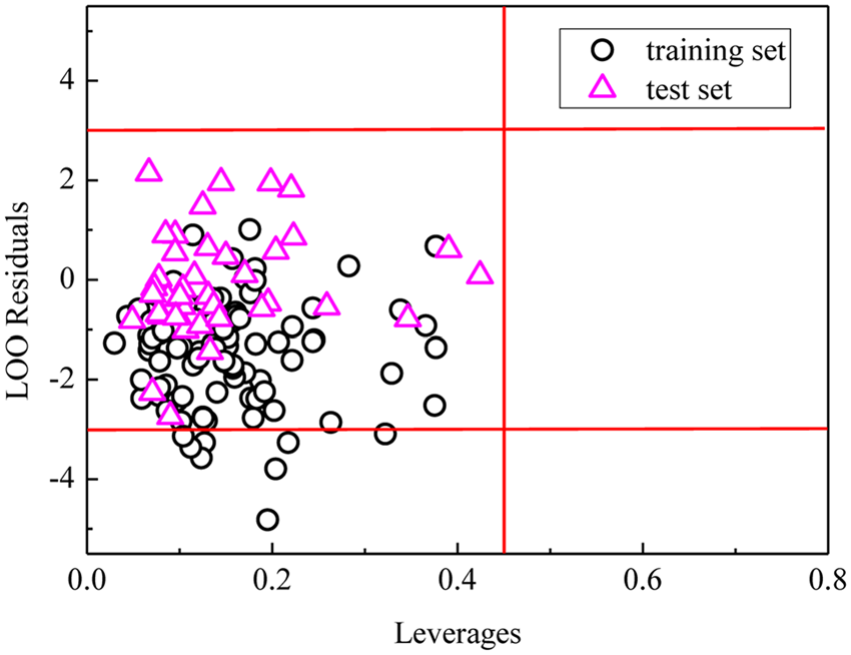
Williams plot of standardized residual *versus* leverage.

In this paper, leverage method was used to define the application domain of the prediction model^27^, which was shown as Williams plot in Fig. 4. Due to many uncertainties in biological experiments, the range of reliable data could be allowed to reach 3 times of standard deviation. In this figure, the application domain is established as a squared area within ± 3 standard deviations and a leverage threshold h* of 3 × 14/91 = 0.46. From Fig. 4, most black circles (they represent compounds in the training set) are located in the middle-left and lower-left region while the magenta triangles (test set samples) are all in the middle-left. This distribution means the leverages of all data are acceptable, and only a small part of the training set data is outside the limit of 3 standard deviations. These training compounds may cause modeling results worse and must be employed carefully. Fortunately, they are in the training set and may not affect model quality significantly. It can be illustrated that this SVM model has good prediction ability to the compounds in test set.

## Discussion

According to the results shown above, based on GA variable selection, 14 variables were picked out to build the final prediction model. The metabolite concentrations oscillate during different phases of life. For example, during the life cycle of a yeast cell the amplitude of metabolite oscillation is usually within 10-fold, with a median of 2.4-fold^28^. Therefore, if the prediction error of –logC is less than 1, it may be accepted in predicting intracellular metabolite concentration. In the earlier study by Zhu and his coworkers^8^, the model has sample size n = 80, R^2^ = 0.683, Q^2^ = 0.59, RMSE = 0.729, as in Table 5. On the other hand, our SVM model has more training set samples n = 91, and good fitting ability with R^2^ = 0.740, Q^2^ = 0.57, RMSE = 0.730. Furthermore, our model was validated by predicting an independent test set containing 39 metabolites, and resulted in R^2^_p_ = 0.586, Q^2^_p_ = 0.58, RMSE_p_ = 0.702.

When randomly separated the metabolites into the training sets and test sets, there may be some overlaps of metabolites in both *E. coli* and *S. cerevisiae*. In order to evaluate the possible influence from same metabolites in both training and test sets, we reconstructed the models with a non-overlap strategy that the same molecules from *E. coli* and *S. cerevisiae* were divided both into the training set, or both into the test set, so as to prevent possible similarity between the training set and the test set. As shown in the Table 6, the reconstructed model kept comparable good performances both in the training set and in the test set, indicating that the evaluation performance is not merely due to training-test-set similarity.

**Table 6:** Comparison of performances between the non-overlap and the random strategy.

Separation Strategy	Training set					Test set		
	R^2^	10-fold		LOO		R^2^_p_	RMSE_p_	Q^2^_p_
		RMSE	Q^2^	RMSE	Q^2^			
non-overlap	0.77	0.72	0.57	0.71	0.59	0.55	0.74	0.54
random	0.74	0.74	0.55	0.73	0.57	0.59	0.70	0.58

In addition, compared with Zhu’s model, our model has a strong advantage in generalizability to different organisms. It is well known that the distributions of metabolite concentrations in different species are quite different, so they are difficult to be completely determined by the degree of metabolic networks only. Zhu’s model was only based on the dataset of *E. coli*, so their model may only fit well for this species. When the 130 compounds in the data sets including both *E. coli* and *S. cerevisiae* were put into one model together using Zhu’s modeling method, the Q^2^ of the reconstructed model could only achieve 0.46 in training set and 0.44 in test set, both less than 0.50. Our SVM model results in Q^2^ as 0.57 for the training set and 0.58 for predicting the test set, that is, greater power in fitness and reduced predictive error.

Obviously, the performance of Zhu’s model could be explained well mainly by one network topological parameter, Degree. Beside Degree, Clustering-Coefficient is another important topological variable, which represents the density of metabolic reactions around the specific metabolites. In our predictive model, Clustering-Coefficient was found to also give some effective information of the differences between the distributions of metabolites’ concentrations in different organisms, and may help to improve the performance of the predictive model.

Furthermore, in this paper metabolic pathway variables were employed to predict metabolite concentration and to explain the complex relationship between the intracellular metabolite concentrations and molecular physicochemical properties. In the evolution, energy metabolism, synthesis and decomposition pathways are most critical. The metabolites in these core pathways generally have higher polarity to reach relative higher concentrations in the hydrophilic environment of plasma. However, some other intracellular metabolic pathways involving different hydrophilic environment, such as signal transduction pathways and the pathways of degradation of harmful substances in the environment, are also important for organisms to adjust the metabolism to external environment. In these non-core pathways, metabolites still need a relatively high concentration to maintain their biological function, despite their lower polarity. For example, in signal transduction, metabolites need lower polarity to pass through the phospholipid bilayer^24, 29, 30^. Therefore, as shown in Fig. 2 and Table 4, our results indicated that the positive correlation between polarity and concentration of metabolites is not strictly linear, and MPF descriptor could be an important variable to help explain this deviation. With the metabolic pathway variables considered, our SVM model may better support for the development of metabolic engineering and synthetic biology.

## Materials and Methods

**Figure 5:**
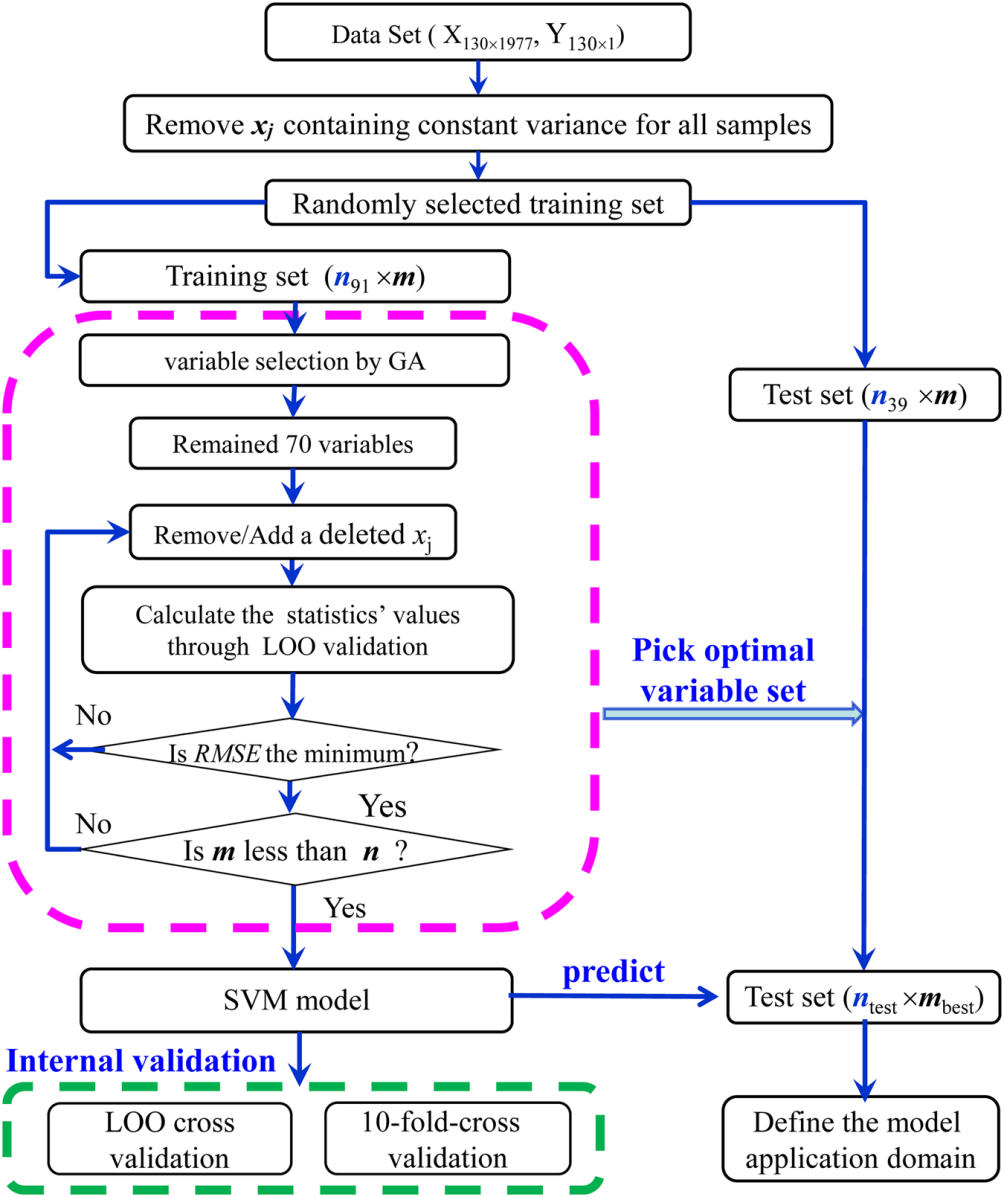
Flow chart of the model development.

The procedure to develop the models between the metabolite intracellular concentrations and the descriptors includes data collection, metabolic network reconstruction, descriptor calculations, variable selections, model development and validation, and test on application domain, as shown in Fig. 5.

### Metabolic network reconstruction of organisms

Metabolic network of organisms was reconstructed by Python-2.7 scripts^30–32^. In the network reconstruction, the metabolic network was automatically reconstructed based on the reactant pairs from KEGG reaction database^33^, which were screened by two criteria. Firstly, reactant pairs which did not appear in the metabolic pathway of the organism should be deleted. Secondly, reactant pairs containing metabolites with unspecified residues were deleted^31, 32^. In the reconstructed metabolic network, each node represents a metabolite while each edge represents a substrate-product relation, which was introduced in details by Ma *et al*.^32^. The reconstructed metabolic network of *E. coli* was consisted of 1002 nodes (metabolites) and 1424 undirectional edges (substrate-product relations), while the one of *S. cerevisiae* was consisted of 2563 nodes (metabolites) and 3123 undirectional edges (substrate-product relations).

### Descriptors calculation

The metabolic network topology parameters were calculated by Network Analyzer Plugin in Cytoscape-3.3.0^34, 35^. And 1850 molecular descriptors were calculated by E-DRAGON^36–38^. These molecular descriptors involve different categories including topological, geometrical, electrostatic and other physicochemical molecular properties of the metabolites.

Pathway variables were chosen as another type of descriptors work to describe the involvement of the metabolites in specific metabolic pathways. There are 116 pathways in *E. coli* and *S. cerevisiae* in the KEGG pathway database^33^. If one metabolite presents in one of these metabolic pathways, its variable for this pathway is defined as 1, otherwise it is recorded as 0. After removing pathway variables that have the same values for almost all metabolites in our dataset described below, the remaining variable set consists of 81 pathway variables which are subject to further variable selection as below. The metabolic pathway variables remaining after variable selection were used to define a new variable, named as MPF (Metabolite Pathways’ Feature) descriptor. If one metabolite participated in any one reaction among the metabolic pathways corresponding to the selected pathway variables, the MPF descriptor of this compound was defined as 1, otherwise it is defined as 0.

### Variable selection

Variable selection was performed prior to the construction of the predictive model in order to eliminate redundant variables and pick out the optimal ones^39^. Due to the absence of some type of atoms in all the metabolites of our dataset, some molecular descriptors’ values might be zero or a constant for all molecules, and these descriptors were abandoned. Then the descriptors with a standard deviation of <0.001 should also be abandoned because of too little statistical meaning. After the preprocessing procedures, 1669 descriptors were retained from the 1977 descriptors.

The 130 metabolites are randomly separated into a training set of 91 samples and a test set of 39 samples^40^. The confidence intervals of model results are calculated with 20 times.

Then genetic algorithm (GA)^13^ is used for variable selection for the training set. Three rounds of selections by GA were executed. In each round of variable selections, the population size of each generation is 100, the maximum generation is 100, and the mutation rate is 0.01, and the optimization objective is RMSE (Equation 3) of each individual in LOO cross-validation. Genetic algorithm was performed by applying the genalg package in R-3.30. After this step, 70 descriptors were retained.

3 $${\rm{RMSE}}=\,\sqrt{\frac{1}{n}{\sum }^{}{({Y}_{exp}-{Y}_{pred})}^{2}}$$

An additional optimization procedure^41^ is applied to filter out irrelevant variables from the 70 descriptors for the training set. In this method, one descriptor which had been deleted from the original 1669 descriptor set was added back into the 70-variables set to construct a new (70 + 1)-variables set, or one descriptor was deleted from these 70 descriptors to obtain a new (70 − 1)-variables set. When one descriptor was added to or deleted from the variable set, the effect of the change was evaluated according to the *RMSE* (Equation 3) obtained in the LOO cross-validation^42^.

If the change in variable set leads to less *RMSE*, the change will be accepted, otherwise it will be rejected, until the minimum RMSE emerged. Then, the variable set in the final model with minimum *RMSE* is extracted as the optimal modeling variable set^41^.

### Model development and validation

A model between the transformed metabolite concentration values(Y = –logC) and the optimal set of descriptors for the 91 samples in the training set was developed by the support vector machine (SVM) regression method, using the kernlab package in R^43^, including model training, evaluating and predicting. Radial basis kernel function exp {−γ|μ − ν|^2^} was chosen to construct a ε-SVR model. The parameters were trained by using grid search over default parameter ranges and the best parameters were obtained as follows: gamma = 0.01, epsilon = 0.20, cost = 11.

The model is internally validated by two methods, including LOO cross-validation and 10-fold cross-validation. In these two methods, the squared cross-validation correlation coefficient (*Q* ^2^) (Equation 4) is employed for the cross-validation^16, 44^.

4 $${Q}^{2}=1-\,\frac{{\sum }^{}{({Y}_{exp}-{Y}_{pred})}^{2}}{{\sum }^{}{({Y}_{exp}-{Y}_{mean})}^{2}}$$

The model developed from the training set was then externally validate by predicting the –logC values of the 39 metabolites in the test set. And in addition, 11 metabolites from *Bacillus subtilis* ^26^ were hired as a new test set to execute an external validation. The predictive correlation coefficients (Q^2^_p_) and RMSE (RMSE_p_) are employed to evaluate the predictive power of the model.

### Application domain

The Williams plot for the SVM model is defined by leverage^45–47^, *hi* (Equation 5), to illustrate the model’s application domain.

5 $${{\rm{h}}}_{i}=\,{x}_{i}^{T}{({X}^{T}X)}^{-1}{x}_{i}\,(i=1,\ldots ,n)$$

where *X* is the *n* × *k* matrix of *k* variable values for *n* training set metabolites, and *x*_*i*_ is the *i*th row vector of *X*. The superscript “*T*” refers to the matrix/vector transpose. The control leverage h* is set as 3*k*/*n*.

## Conclusions

Combining chemical descriptors, topological parameters and metabolic pathways descriptors, a machine learning model can be constructed to predict the metabolite concentration with relative reliability. 14 optimal descriptors are effectively derived from a great amount of DRAGON descriptors and metabolic pathways descriptors according to Q^2^ and RMSE values by the GA variable selection procedures. These descriptors were significant in construction of a SVM regression model, based on a data set of 130 metabolites of the *E. coli* and *S. cerevisiae*, which was randomly separated into a training set of 91 samples and a test set of 39 samples. The results of internal LOO and 10-fold cross-validation indicated that the model is robust, while the external validations on the test set showed good prediction powers. Therefore, this SVM model might be useful for prediction of the intracellular metabolites concentration with a well-defined application domain when experimental values are difficult to be acquired.

## Electronic supplementary material

Supplementary information
