# Supplementary material for: Theoretical Studies of Intracellular Concentration of Micro-organisms’ Metabolites
Source: Sci Rep. 2017 Aug 22;7:9048. doi: 10.1038/s41598-017-08793-2 (PMC5567373; doi:10.1038/s41598-017-08793-2)
Supplement: Supplementary file 1 — Supplementary information [file 41598_2017_8793_MOESM1_ESM.pdf]

Theoretical Studies of Intracellular Concentration of Micro-organisms' Metabolites

Hai-Feng Yang [1], Xiao-Nan Zhang[2], Yan Li [3], Yong-Hong Zhang[4]*, Qin Xu[1]*, and Dong-Qing Wei[1]

[1] State Key Laboratory of Microbial Metabolism, and College of Life Sciences and Biotechnology, Shanghai Jiao Tong University, Shanghai, China;

[2] Chongqing key Laboratory of Oral Diseases and Biomedical Sciences, Chongqing Municipal Key Laboratory of Oral Biomedical Engineering of Higher Education, and College of Stomatology, Chongqing Medical University, Chongqing, China

[3] Department of Chinese Traditional Medicine, Chongqing Medical University, Chongqing, China

[4] Medicine Engineering Research Center, and College of Pharmacy, Chongqing Medical University, Chongqing, China

* Correspondence: zhyvonne26@126.com; xuqin523@sjtu.edu.cn; Tel.: +86-21-34204348 Fax: +86-21-34204573.

Supplementary information

Table S1. Information of selected pathways in the dataset.

Organism	Metabolite	KEGG ID	Map 00625	Map 00626	Map 02020	Map 03070	Map 04122	MPF	Concentration
E.coli	ATP	C00002	0	0	0	1	0	1	9.63E-03
	NAD	C00003	0	0	0	0	0	0	2.55E-03
	NADH	C00004	0	0	0	0	0	0	8.32E-05
	ADP	C00008	0	0	0	0	0	0	5.55E-04
	Coenzyme-A	C00010	0	0	0	0	0	0	1.37E-03
	UDP	C00015	0	0	0	0	0	0	1.79E-03
	FAD	C00016	0	0	0	0	0	0	1.73E-04
	S-adenosyl-L-methionine	C00019	0	0	0	0	1	1	1.84E-04
	AMP	C00020	0	0	0	0	0	0	2.81E-04
	Acetyl-CoA	C00024	1	0	0	0	0	1	6.06E-04
	Glutamate	C00025	0	0	1	0	0	1	9.60E-02
	α-Ketoglutarate	C00026	0	0	0	0	0	0	4.43E-04
	UDP-glucose	C00029	0	0	0	0	0	0	2.50E-03
	GDP	C00035	0	0	0	0	0	0	6.76E-04
	Alanine	C00041	0	0	0	0	1	1	2.55E-03
	Succinate	C00042	1	0	0	0	0	1	5.69E-04
	UDP-N-acetyl-glucosamine	C00043	0	0	0	0	0	0	9.24E-03

	GTP	C00044	0	0	0	0	1	1	4.87E-03
	Lysine	C00047	0	0	0	0	0	0	4.05E-04
	Aspartate	C00049	0	1	0	0	0	1	4.23E-03
	Glutathione	C00051	0	0	0	0	0	0	1.66E-02
	CMP	C00055	0	0	0	0	0	0	3.60E-04
	Flavin mononucleotide	C00061	0	0	0	0	0	0	5.37E-05
	Arginine	C00062	0	0	0	0	0	0	5.69E-04
	CTP	C00063	0	0	0	0	0	0	2.73E-03
	Glutamine	C00064	0	0	1	0	0	1	3.81E-03
	Serine	C00065	0	0	0	0	0	0	6.80E-05
	Methionine	C00073	0	0	0	0	0	0	1.45E-04
	Phosphoenolpyruvate	C00074	0	0	0	0	0	0	1.84E-04
	UTP	C00075	0	0	0	0	0	0	8.29E-03
	Ornithine	C00077	0	0	0	0	0	0	1.01E-05
	Tryptophan	C00078	0	0	0	0	0	0	1.21E-05
	Phenylalanine	C00079	0	0	0	0	0	0	1.82E-05
	Inosine-triphosphate	C00081	0	0	0	0	0	0	2.05E-04
	Tyrosine	C00082	0	0	0	0	0	0	2.89E-05
	Malonyl-CoA	C00083	0	0	0	0	0	0	3.54E-05
	Succinyl-CoA	C00091	0	1	0	0	0	1	2.33E-04
	Propionyl-CoA	C00100	0	0	0	0	0	0	5.32E-06
	Inosine-diphosphate	C00104	0	0	0	0	0	0	2.38E-05
	Anthranilate	C00108	0	0	0	0	0	0	3.48E-06
	Dihydroxyacetonephosphate	C00111	0	0	0	0	0	0	3.74E-04
	PRPP	C00119	0	0	0	0	0	0	2.58E-04
	Fumarate	C00122	0	0	1	0	0	1	1.15E-04
	Glutathione disulfide	C00127	0	0	0	0	0	0	2.37E-03
	Inosine-monophosphate	C00130	0	0	0	0	0	0	2.72E-04
	dATP	C00131	0	0	0	0	0	0	1.55E-05
	Histidine	C00135	0	0	0	0	0	0	6.76E-05
	Myo-inositol	C00137	0	0	0	0	0	0	5.72E-06
	GMP	C00144	0	0	0	0	0	0	2.37E-05
	Adenine	C00147	0	0	0	0	0	0	1.47E-06
	Proline	C00148	0	0	0	0	0	0	3.85E-04
	Malate	C00149	0	0	1	0	0	1	1.68E-03
	Asparagine	C00152	0	0	0	0	0	0	5.11E-04
	Homocysteine	C00155	0	0	0	0	0	0	3.70E-04
	4-Hydroxybenzoate	C00156	0	0	0	0	0	0	5.22E-05
	Citrate	C00158	0	0	1	0	0	1	1.96E-03

	Phenylpyruvate	C00166	0	0	0	0	0	0	8.98E-05
	UDP-glucaronate	C00167	0	0	0	0	0	0	5.66E-04
	Valine	C00183	0	0	0	0	0	0	4.02E-03
	Threonine	C00188	0	0	0	0	0	0	1.79E-04
	2,3-Dihydroxybenzoic acid	C00196	0	0	0	0	0	0	1.38E-04
	3-Phosphoglycerate	C00197	0	0	0	0	0	0	1.54E-03
	Adenosine	C00212	0	0	0	0	0	0	1.31E-07
	Adenosine-phosphosulfate	C00224	0	0	0	0	0	0	6.63E-06
	Acetyl phosphate	C00227	0	0	0	0	0	0	1.07E-03
	Guanine	C00242	0	0	0	0	0	0	1.88E-04
	Gluconate	C00257	0	0	0	0	0	0	4.16E-05
	Glycerate	C00258	0	0	0	0	0	0	1.41E-03
	Uridine	C00299	0	0	0	0	0	0	2.09E-03
	Citrulline	C00327	0	0	0	0	0	0	1.35E-03
	Deoxyguanosine	C00330	0	0	0	0	0	0	5.22E-07
	Acetoacetyl-CoA	C00332	0	0	0	0	0	0	2.18E-05
	Dihydroorotate	C00337	0	0	0	0	0	0	1.19E-05
	6-phospho-D-gluconate	C00345	0	0	0	0	0	0	3.77E-03
	Glucosamine-6_phosphate	C00352	0	0	0	0	0	0	1.15E-03
	Fructose-1,6-bisphosphate	C00354	0	0	0	0	0	0	1.52E-02
	dAMP	C00360	0	0	0	0	0	0	8.84E-06
	dGMP	C00362	0	0	0	0	0	0	5.07E-05
	dTDP	C00363	0	0	0	0	0	0	3.78E-04
	Cytosine	C00380	0	0	0	0	0	0	1.41E-05
	Guanosine	C00387	0	0	0	0	0	0	1.62E-06
	Aconitate	C00417	0	0	0	0	0	0	1.61E-05
	N-acetyl-ornithine	C00437	0	0	0	0	0	0	4.33E-05
	Carbamyl-aspartate	C00438	0	0	0	0	0	0	5.90E-04
	dCTP	C00458	0	0	0	0	0	0	3.45E-05
	dTTP	C00459	0	0	0	0	0	0	4.62E-03
	Cytidine	C00475	0	0	0	0	0	0	2.59E-06
	Shikimate	C00493	0	0	0	0	0	0	1.41E-05
	ADP-glucose	C00498	0	0	0	0	0	0	4.27E-06
	Deoxyadenosine	C00559	0	0	0	0	0	0	2.82E-06
	Cyclic-AMP	C00575	0	0	0	0	0	0	3.52E-05
	Histidinol	C00860	0	0	0	0	0	0	1.28E-05
	Quinolinate	C03722	0	0	0	0	0	0	1.15E-05
S.cerevisiae	ATP	C00002	0	0	0	1	0	1	3.39E-03
	NAD+	C00003	0	0	0	0	0	0	4.70E-04

	NADH	C00004	0	0	0	0	0	0	4.50E-04
	ADP	C00008	0	0	0	0	0	0	6.30E-04
	AMP	C00020	0	0	0	0	0	0	1.60E-04
	Pyruvate	C00022	0	0	0	0	0	0	1.24E-03
	L-Glutamate	C00025	0	0	1	0	0	1	8.18E-02
	2-Oxoglutarate	C00026	0	0	0	0	0	0	1.11E-03
	GDP	C00035	0	0	0	0	0	0	2.20E-04
	L-Alanine	C00041	0	0	0	0	1	1	2.47E-02
	Succinate	C00042	1	0	0	0	0	1	3.40E-04
	GTP	C00044	0	0	0	0	1	1	5.90E-04
	L-Aspartate	C00049	0	1	0	0	0	1	1.57E-02
	L-Arginine	C00062	0	0	0	0	0	0	3.58E-02
	L-Glutamine	C00064	0	0	1	0	0	1	8.04E-02
	Phosphoenolpyruvate	C00074	0	0	0	0	0	0	1.90E-04
	L-Tryptophan	C00078	0	0	0	0	0	0	3.00E-05
	L-Phenylalanine	C00079	0	0	0	0	0	0	3.50E-04
	L-Tyrosine	C00082	0	0	0	0	0	0	1.60E-04
	D-Fructose 6-phosphate	C00085	0	0	0	0	0	0	3.20E-04
	D-Glucose 6-phosphate	C00092	0	0	1	0	0	1	3.70E-03
	sn-Glycerol 3-phosphate	C00093	0	0	0	0	0	0	5.00E-04
	D-Glucose 1-phosphate	C00103	0	0	0	0	0	0	9.00E-05
	Glycerone phosphate	C00111	0	0	0	0	0	0	7.20E-04
	D-Ribose 5-phosphate	C00117	0	0	0	0	0	0	4.10E-04
	Fumarate	C00122	0	0	1	0	0	1	1.65E-03
	GMP	C00144	0	0	0	0	0	0	6.00E-05
	Adenine	C00147	0	0	0	0	0	0	5.00E-05
	Malate	C00149	0	0	1	0	0	1	1.68E-03
	L-Asparagine	C00152	0	0	0	0	0	0	4.66E-03
	Citrate	C00158	0	0	1	0	0	1	1.47E-03
	3-Phospho-D-glycerate	C00197	0	0	0	0	0	0	8.90E-04
	D-Ribulose 5-phosphate	C00199	0	0	0	0	0	0	2.00E-05
	Isocitrate	C00311	0	0	0	0	0	0	1.90E-04
	6-Phospho-D-gluconate	C00345	0	0	0	0	0	0	8.70E-04
	D-Fructose 1,6-bisphosphate	C00354	0	0	0	0	0	0	8.76E-03
	Pantothenate	C00864	0	0	0	0	0	0	2.10E-04

Table S2. Information of selected pathways in training set and test set.

	Organism	Metabolite	KEGG ID	Map 00625	Map 00626	Map 02020	Map 03070	Map 04122	MPF
Training Set	E.coli	ATP	C00002	0	0	0	1	0	1
		NAD	C00003	0	0	0	0	0	0
		NADH	C00004	0	0	0	0	0	0
		ADP	C00008	0	0	0	0	0	0
		Coenzyme-A	C00010	0	0	0	0	0	0
		UDP	C00015	0	0	0	0	0	0
		FAD	C00016	0	0	0	0	0	0
		AMP	C00020	0	0	0	0	0	0
		Acetyl-CoA	C00024	1	0	0	0	0	1
		Glutamate	C00025	0	0	1	0	0	1
		α-Ketoglutarate	C00026	0	0	0	0	0	0
		UDP-glucose	C00029	0	0	0	0	0	0
		Alanine	C00041	0	0	0	0	1	1
		UDP-N-acetyl-glucosamine	C00043	0	0	0	0	0	0
		GTP	C00044	0	0	0	0	1	1
		lysine	C00047	0	0	0	0	0	0
		Aspartate	C00049	0	1	0	0	0	1
		Glutathione	C00051	0	0	0	0	0	0
		CMP	C00055	0	0	0	0	0	0
		Flavin mononucleotide	C00061	0	0	0	0	0	0
		Arginine	C00062	0	0	0	0	0	0
		CTP	C00063	0	0	0	0	0	0
		Methionine	C00073	0	0	0	0	0	0
		Phosphoenol- pyruvate	C00074	0	0	0	0	0	0
		UTP	C00075	0	0	0	0	0	0
		Ornithine	C00077	0	0	0	0	0	0
		Inosine-triphosphate	C00081	0	0	0	0	0	0
		Tyrosine	C00082	0	0	0	0	0	0
		Malonyl-CoA	C00083	0	0	0	0	0	0
		Succinyl-CoA	C00091	0	1	0	0	0	1
		Propionyl-CoA	C00100	0	0	0	0	0	0
		Inosine-diphosphate	C00104	0	0	0	0	0	0
		Anthranilate	C00108	0	0	0	0	0	0
		Fumarate	C00122	0	0	1	0	0	1
		Inosine-monophosphate	C00130	0	0	0	0	0	0

		GMP	C00144	0	0	0	0	0	0
		Adenine	C00147	0	0	0	0	0	0
		Proline	C00148	0	0	0	0	0	0
		Malate	C00149	0	0	1	0	0	1
		Asparagine	C00152	0	0	0	0	0	0
		Homocysteine	C00155	0	0	0	0	0	0
		Citrate	C00158	0	0	1	0	0	1
		Phenylpyruvate	C00166	0	0	0	0	0	0
		UDP-glucaronate	C00167	0	0	0	0	0	0
		2,3-Dihydroxybenzoic acid	C00196	0	0	0	0	0	0
		Adenosine	C00212	0	0	0	0	0	0
		Acetyl phosphate	C00227	0	0	0	0	0	0
		Gluconate	C00257	0	0	0	0	0	0
		Glycerate	C00258	0	0	0	0	0	0
		Uridine	C00299	0	0	0	0	0	0
		Citrulline	C00327	0	0	0	0	0	0
		Acetoacetyl-CoA	C00332	0	0	0	0	0	0
		Dihydroorotate	C00337	0	0	0	0	0	0
		6-phospho-D-gluconate	C00345	0	0	0	0	0	0
		Glucosamine-6_phosphate	C00352	0	0	0	0	0	0
		Fructose-1,6-bisphosphate	C00354	0	0	0	0	0	0
		dAMP	C00360	0	0	0	0	0	0
		dGMP	C00362	0	0	0	0	0	0
		Cytosine	C00380	0	0	0	0	0	0
		Guanosine	C00387	0	0	0	0	0	0
		Aconitate	C00417	0	0	0	0	0	0
		N-acetyl-ornithine	C00437	0	0	0	0	0	0
		Carbamyl-aspartate	C00438	0	0	0	0	0	0
		Cytidine	C00475	0	0	0	0	0	0
		Shikimate	C00493	0	0	0	0	0	0
		ADP-glucose	C00498	0	0	0	0	0	0
		Cyclic-AMP	C00575	0	0	0	0	0	0
		Histidinol	C00860	0	0	0	0	0	0
		Quinolinate	C03722	0	0	0	0	0	0
	S.cerevisiae	NADH	C00004	0	0	0	0	0	0
		ADP	C00008	0	0	0	0	0	0
		AMP	C00020	0	0	0	0	0	0
		Pyruvate	C00022	0	0	0	0	0	0
		Succinate	C00042	1	0	0	0	0	1

		L-Arginine	C00062	0	0	0	0	0	0
		L-Glutamine	C00064	0	0	1	0	0	1
		Phosphoenolpyruvate	C00074	0	0	0	0	0	0
		L-Tryptophan	C00078	0	0	0	0	0	0
		L-Phenylalanine	C00079	0	0	0	0	0	0
		L-Tyrosine	C00082	0	0	0	0	0	0
		D-Fructose 6-phosphate	C00085	0	0	0	0	0	0
		D-Glucose 6-phosphate	C00092	0	0	1	0	0	1
		sn-Glycerol-3-phosphate	C00093	0	0	0	0	0	0
		Glycerone phosphate	C00111	0	0	0	0	0	0
		Fumarate	C00122	0	0	1	0	0	1
		GMP	C00144	0	0	0	0	0	0
		Adenine	C00147	0	0	0	0	0	0
		Malate	C00149	0	0	1	0	0	1
		Citrate	C00158	0	0	1	0	0	1
		6-Phospho-D-gluconate	C00345	0	0	0	0	0	0
		D-Fructose-1,6-bisphosphate	C00354	0	0	0	0	0	0
Test Set	E.coli	S-adenosyl-L-methionine	C00019	0	0	0	0	1	1
		GDP	C00035	0	0	0	0	0	0
		Succinate	C00042	1	0	0	0	0	1
		Glutamine	C00064	0	0	1	0	0	1
		Serine	C00065	0	0	0	0	0	0
		Tryptophan	C00078	0	0	0	0	0	0
		Phenylalanine	C00079	0	0	0	0	0	0
		Dihydroxy-acetonephosphate	C00111	0	0	0	0	0	0
		PRPP	C00119	0	0	0	0	0	0
		Glutathione disulfide	C00127	0	0	0	0	0	0
		dATP	C00131	0	0	0	0	0	0
		Histidine	C00135	0	0	0	0	0	0
		Myo-inositol	C00137	0	0	0	0	0	0
		4-Hydroxybenzoate	C00156	0	0	0	0	0	0
		Valine	C00183	0	0	0	0	0	0
		Threonine	C00188	0	0	0	0	0	0
		3-Phosphoglycerate	C00197	0	0	0	0	0	0
		Adenosine-phosphosulfate	C00224	0	0	0	0	0	0
		Guanine	C00242	0	0	0	0	0	0
		Deoxyguanosine	C00330	0	0	0	0	0	0
		dTDP	C00363	0	0	0	0	0	0
		dCTP	C00458	0	0	0	0	0	0

		dTTP	C00459	0	0	0	0	0	0
		Deoxyadenosine	C00559	0	0	0	0	0	0
	S.cerevisiae	ATP	C00002	0	0	0	1	0	1
		NAD+	C00003	0	0	0	0	0	0
		L-Glutamate	C00025	0	0	1	0	0	1
		2-Oxoglutarate	C00026	0	0	0	0	0	0
		GDP	C00035	0	0	0	0	0	0
		L-Alanine	C00041	0	0	0	0	1	1
		GTP	C00044	0	0	0	0	1	1
		L-Aspartate	C00049	0	1	0	0	0	1
		D-Glucose 1-phosphate	C00103	0	0	0	0	0	0
		D-Ribose 5-phosphate	C00117	0	0	0	0	0	0
		L-Asparagine	C00152	0	0	0	0	0	0
		3-Phospho-D-glycerate	C00197	0	0	0	0	0	0
		D-Ribulose 5-phosphate	C00199	0	0	0	0	0	0
		Isocitrate	C00311	0	0	0	0	0	0
		Pantothenate	C00864	0	0	0	0	0	0

Table S3. Information of predicted and measured concentrations in training set and test set.

	Organism	Metabolite	KEGG ID	Ce	Cp	Error
Training Set	E.coli	ATP	C00002	2.02	2.31	-0.29
		NAD	C00003	2.59	2.82	-0.22
		NADH	C00004	4.08	3.86	0.22
		ADP	C00008	3.26	3.92	-0.67
		Coenzyme-A	C00010	2.86	2.64	0.22
		UDP	C00015	2.75	2.53	0.22
		FAD	C00016	3.76	3.98	-0.22
		AMP	C00020	3.55	3.75	-0.20
		Acetyl-CoA	C00024	3.22	3.44	-0.22
		Glutamate	C00025	1.02	1.27	-0.26
		α-Ketoglutarate	C00026	3.35	3.47	-0.12
		UDP-glucose	C00029	2.60	3.02	-0.41
		Alanine	C00041	2.59	2.37	0.22
		UDP-N-acetyl-glucosamine	C00043	2.03	3.10	-1.06
		GTP	C00044	2.31	2.09	0.22
		lysine	C00047	3.39	3.62	-0.22
		Aspartate	C00049	2.37	2.15	0.22
		Glutathione	C00051	1.78	2.00	-0.22
		CMP	C00055	3.44	3.52	-0.07
		Flavin mononucleotide	C00061	4.27	4.05	0.22
		Arginine	C00062	3.24	3.02	0.22
		CTP	C00063	2.56	2.57	-0.01
		Methionine	C00073	3.84	3.62	0.22
		Phosphoenolpyruvate	C00074	3.74	3.62	0.12
		UTP	C00075	2.08	2.36	-0.27
		Ornithine	C00077	5.00	3.43	1.56
		Inosine-triphosphate	C00081	3.69	3.47	0.22
		Tyrosine	C00082	4.54	4.27	0.27
		Malonyl-CoA	C00083	4.45	4.23	0.22
		Succinyl-CoA	C00091	3.63	3.45	0.18
		Propionyl-CoA	C00100	5.27	4.67	0.60
		Inosine-diphosphate	C00104	4.62	3.78	0.85
		Anthranilate	C00108	5.46	4.85	0.61
		Fumarate	C00122	3.94	3.72	0.22
		Inosine-monophosphate	C00130	3.57	3.81	-0.25

		GMP	C00144	4.63	3.82	0.80
		Adenine	C00147	5.83	5.47	0.37
		Proline	C00148	3.41	3.64	-0.22
		Malate	C00149	2.77	2.77	0.01
		Asparagine	C00152	3.29	3.51	-0.22
		Homocysteine	C00155	3.43	3.63	-0.20
		Citrate	C00158	2.71	2.54	0.17
		Phenylpyruvate	C00166	4.05	4.27	-0.22
		UDP-glucaronate	C00167	3.25	3.23	0.02
		2,3-Dihydroxybenzoic acid	C00196	3.86	5.18	-1.32
		Adenosine	C00212	6.88	5.73	1.16
		Acetyl phosphate	C00227	2.97	3.24	-0.27
		Gluconate	C00257	4.38	3.99	0.39
		Glycerate	C00258	2.85	3.75	-0.89
		Uridine	C00299	2.68	4.16	-1.48
		Citrulline	C00327	2.87	3.32	-0.45
		Acetoacetyl-CoA	C00332	4.66	4.44	0.22
		Dihydroorotate	C00337	4.92	4.74	0.19
		6-phospho-D-gluconate	C00345	2.42	2.77	-0.35
		Glucosamine-6_phosphate	C00352	2.94	3.64	-0.70
		Fructose-1,6-bisphosphate	C00354	1.82	2.04	-0.22
		dAMP	C00360	5.05	4.75	0.30
		dGMP	C00362	4.29	4.30	-0.01
		Cytosine	C00380	4.85	4.26	0.59
		Guanosine	C00387	5.79	5.18	0.61
		Aconitate	C00417	4.79	4.46	0.33
		N-acetyl-ornithine	C00437	4.36	3.21	1.16
		Carbamyl-aspartate	C00438	3.23	3.71	-0.48
		Cytidine	C00475	5.59	4.84	0.75
		Shikimate	C00493	4.85	4.63	0.22
		ADP-glucose	C00498	5.37	4.61	0.76
		Cyclic-AMP	C00575	4.45	4.68	-0.22
		Histidinol	C00860	4.89	4.63	0.26
		Quinolinate	C03722	4.94	4.72	0.22
	S.cerevisiae	NADH	C00004	3.35	4.09	-0.75
		ADP	C00008	3.20	3.42	-0.22
		AMP	C00020	3.80	3.57	0.22
		Pyruvate	C00022	2.91	2.68	0.22
		Succinate	C00042	3.47	3.25	0.22

		L-Arginine	C00062	1.45	3.02	-1.58
		L-Glutamine	C00064	1.09	2.41	-1.32
		Phosphoenolpyruvate	C00074	3.72	3.62	0.11
		L-Tryptophan	C00078	4.52	4.63	-0.11
		L-Phenylalanine	C00079	3.46	4.41	-0.95
		L-Tyrosine	C00082	3.80	4.33	-0.54
		D-Fructose 6-phosphate	C00085	3.49	3.45	0.04
		D-Glucose 6-phosphate	C00092	2.43	2.48	-0.05
		sn-Glycerol 3-phosphate	C00093	3.30	3.08	0.22
		Glycerone phosphate	C00111	3.14	3.24	-0.10
		Fumarate	C00122	2.78	3.73	-0.95
		GMP	C00144	4.22	3.78	0.44
		Adenine	C00147	4.30	5.59	-1.29
		Malate	C00149	2.77	2.77	0.01
		Citrate	C00158	2.83	2.61	0.22
		6-Phospho-D-gluconate	C00345	3.06	2.84	0.22
		D-Fructose 1,6-bisphosphate	C00354	2.06	2.04	0.02
Test Set	E.coli	S-adenosyl-L-methionine	C00019	3.74	3.68	0.06
		GDP	C00035	3.17	3.21	-0.04
		Succinate	C00042	3.24	3.05	0.20
		Glutamine	C00064	2.42	2.36	0.06
		Serine	C00065	4.17	3.64	0.53
		Tryptophan	C00078	4.92	4.69	0.23
		Phenylalanine	C00079	4.74	4.35	0.39
		Dihydroxyacetonephosphate	C00111	3.43	2.89	0.53
		PRPP	C00119	3.59	3.23	0.36
		Glutathione disulfide	C00127	2.63	2.87	-0.25
		dATP	C00131	4.81	4.19	0.62
		Histidine	C00135	4.17	3.79	0.38
		Myo-inositol	C00137	5.24	4.94	0.30
		4-Hydroxybenzoate	C00156	4.28	4.66	-0.38
		Valine	C00183	2.40	3.59	-1.19
		Threonine	C00188	3.75	3.52	0.23
		3-Phosphoglycerate	C00197	2.81	3.45	-0.63
		Adenosine-phosphosulfate	C00224	5.18	4.66	0.52
		Guanine	C00242	3.73	4.97	-1.24
		Deoxyguanosine	C00330	6.28	5.28	1.00
		dTDP	C00363	3.42	3.29	0.13
		dCTP	C00458	4.46	2.55	1.91

		dTTP	C00459	2.34	3.33	-1.00
		Deoxyadenosine	C00559	5.55	6.04	-0.49
	S.cerevisiae	ATP	C00002	2.47	2.08	0.39
		NAD+	C00003	3.33	2.86	0.46
		L-Glutamate	C00025	1.09	1.52	-0.44
		2-Oxoglutarate	C00026	2.95	3.36	-0.40
		GDP	C00035	3.66	3.21	0.45
		L-Alanine	C00041	1.61	2.42	-0.81
		GTP	C00044	3.23	2.02	1.21
		L-Aspartate	C00049	1.80	2.41	-0.60
		D-Glucose 1-phosphate	C00103	4.05	3.49	0.56
		D-Ribose 5-phosphate	C00117	3.39	3.20	0.18
		L-Asparagine	C00152	2.33	3.80	-1.47
		3-Phospho-D-glycerate	C00197	3.05	3.45	-0.39
		D-Ribulose 5-phosphate	C00199	4.70	3.14	1.56
		Isocitrate	C00311	3.72	3.22	0.50
		Pantothenate	C00864	3.68	3.63	0.05

Table S4 the Information of 14 Descriptors and Metabolite Concentrations

Organism	Metabolite	KEGG ID	Concentration	Clustering Coefficient	Degree	BCUT_SLOGP_2	BCUT_SMR_3	GCUT_PEOE_1	PEOE_VSA+0	PEOE_VSA+5	opr_nring	vsa_hyd	SlogP_VSA9	6mem rings Molecules	ClogP	RPCG	MPF
	ATP	C00002	9.63E-03	0.33333333	4	0.42522323	2.9664838	-0.62666225	8.5307722	0	3	54.13358	50.375816	1	-4.546	0.096	1
	NAD	C00003	2.55E-03	0.2	5	0.54069591	2.9688232	-0.51378423	46.96413	12.949531	5	175.9922	34.699287	2	-7.801	0.063	0
	NADH	C00004	8.32E-05	0.33333333	3	0.54058594	2.9802225	-0.52770728	57.703442	0	5	175.9922	34.699287	2	-4.689	0.07	0
	ADP	C00008	5.55E-04	0.16666667	4	0.46371293	2.9660692	-0.63219053	8.5307722	0	3	54.13358	34.190132	1	-3.886	0.104	0
	coenzyme-A	C00010	1.37E-03	0	30	0.36055136	2.981642	-0.53020346	146.6619	12.949531	3	217.6596	117.02785	1	-3.873	0.064	0
	UDP	C00015	1.79E-03	0.16666667	9	0.46295124	2.9807563	-0.61501384	37.53159	12.949531	2	83.54203	55.724426	1	-3.947	0.109	0
	FAD	C00016	1.73E-04	0	2	0.51087809	2.9666543	-0.52933145	72.55645	12.949531	6	230.1172	130.72704	4	-3.385	0.062	0
	S-adenosyl-L-methionine	C00019	1.84E-04	0	4	0.4655481	3.0290532	-0.36434722	8.5307722	0	3	121.5368	2.3279202	1	-5.076	0.056	1
	AMP	C00020	2.81E-04	0.03676471	17	0.50219971	2.9641578	-0.5823155	8.5307722	0	3	54.13358	18.004448	1	-3.225	0.113	0
	acetyl-CoA	C00024	6.06E-04	0.03571429	8	0.34683856	2.9816422	-0.52860516	178.20573	12.949531	3	266.8286	182.57339	1	-3.539	0.061	1
	glutamate	C00025	9.60E-02	0.02380952	21	0.30460405	2.8003836	-0.59298134	40.471355	0	0	38.31654	0	0	-2.694	0.155	1
	α-ketoglutarate	C00026	4.43E-04	0.0952381	7	0.19902514	2.6908374	-0.66512686	40.471355	0	0	31.61699	0	0	-1.303	0.2	0
	UDP-glucose	C00029	2.50E-03	0.13333333	6	0.54046905	2.9826562	-0.4773497	77.073494	12.949531	3	139.4346	56.233585	2	-4.183	0.077	0
	GDP	C00035	6.76E-04	0.33333333	3	0.42566904	2.9674129	-0.62751305	8.5307722	12.949531	3	54.13358	34.190132	1	-4.87	0.097	0
	alanine	C00041	2.55E-03	0.06666667	6	0.2142	2.6955085	-0.37000412	12.796158	0	0	36.108	33.326015	0	-3.124	0.254	1
	succinate	C00042	5.69E-04	0	7	0.27618599	2.6600556	-0.70726633	46.478542	0	0	31.61699	0	0	-0.526	0.192	1
	UDP-N-acetyl-glucosamine	C00043	9.24E-03	0.06666667	6	0.51742595	2.9923887	-0.47738761	108.61733	12.949531	3	167.0841	89.559601	2	-3.743	0.073	0
	GTP	C00044	4.87E-03	0.07142857	8	0.39961782	2.9678149	-0.62380373	8.5307722	12.949531	3	54.13358	50.375816	1	-5.531	0.091	1
	lysine	C00047	4.05E-04	0	3	0.2142	2.8649819	-0.34771264	34.293629	0	0	74.42454	0	0	-3.424	0.18	0
	aspartate	C00049	4.23E-03	0.07272727	11	0.30306789	2.7412763	-0.64470696	31.940584	0	0	22.50805	0	0	-2.412	0.156	1
	glutathione	C00051	1.66E-02	0.00666667	25	0.40836948	2.856231	-0.48123536	53.672318	0	0	83.10015	0	0	-3.051	0.081	0
	CMP	C00055	3.60E-04	0.16666667	4	0.51310664	2.976644	-0.56484812	25.276688	0	2	83.54203	39.538742	1	-3.262	0.12	0
	flavin mononucleotide	C00061	5.37E-05	0	1	0.49368736	2.9067736	-0.5330255	34.123089	12.949531	3	175.9836	111.70427	3	-1.777	0.098	0
	arginine	C00062	5.69E-04	0	3	0.30467409	2.8467581	-0.37197614	25.762857	43.095066	0	58.61605	0	0	-3.517	0.129	0
	CTP	C00063	2.73E-03	0.33333333	3	0.47429019	2.9785659	-0.6171627	25.276688	0	2	83.54203	71.91011	1	-4.584	0.1	0
	glutamine	C00064	3.81E-03	0.2	5	0.33341646	2.8105457	-0.44240996	34.464172	0	0	38.31654	0	0	-3.375	0.152	1
	serine	C00065	6.80E-05	0	6	0.39069223	2.7133796	-0.44136977	0	0	0	28.75803	0	0	-2.811	0.187	0
	methionine	C00073	1.45E-04	0	4	0.33664057	2.8324771	-0.3317405	38.559017	0	0	91.43773	32.21954	0	-1.731	0.218	0
	phosphoenolpyruvate	C00074	1.84E-04	0	3	0.19482203	2.5316682	-0.68372554	0	0	0	37.86697	47.777321	0	-1.006	0.24	0
	UTP	C00075	8.29E-03	0.33333333	3	0.42419368	2.9810627	-0.61808562	37.53159	12.949531	2	83.54203	71.91011	1	-4.607	0.1	0
	ornithine	C00077	1.01E-05	0	3	0.2142	2.8419199	-0.34579369	34.464172	0	0	58.61605	0	0	-3.953	0.187	0
	tryptophan	C00078	1.21E-05	0	3	0.67442864	2.7640889	-0.40016633	42.886093	0	2	117.1582	0	1	-1.566	0.159	0
	phenylalanine	C00079	1.82E-05	0	3	0.61489522	2.7547119	-0.38270196	17.232086	0	1	103.7591	0	1	-1.556	0.191	0
	inosine-triphosphate	C00081	2.05E-04	0	2	0.46336168	2.9677224	-0.6279754	8.5307722	12.949531	3	54.13358	50.375816	1	-5.495	0.096	0
	tyrosine	C00082	2.89E-05	0	2	0.59373242	2.7553606	-0.43409511	17.232086	0	1	96.40908	5.2434282	1	-2.223	0.162	0
	malonyl-CoA	C00083	3.54E-05	0	1	0.35369551	2.9816422	-0.52977639	169.90117	12.949531	3	253.2287	149.24739	1	-4.171	0.057	0
	succinyl-CoA	C00091	2.33E-04	0	3	0.35369551	2.9816422	-0.52941835	187.13326	12.949531	3	269.0371	149.24739	1	-3.941	0.057	1
	propionyl-CoA	C00100	5.32E-06	0	6	0.34683856	2.9816422	-0.52452463	176.69014	12.949531	3	282.6371	182.57339	1	-3.01	0.061	0
	inosine-diphosphate	C00104	2.38E-05	0	1	0.50189865	2.9673195	-0.63032854	8.5307722	12.949531	3	54.13358	34.190132	1	-4.834	0.103	0
	anthranilate	C00108	3.48E-06	0	2	0.68959385	2.5211341	-0.59265256	8.9080906	0	1	72.14207	2.7567475	1	1.208	0.207	0
	dihydroxyacetonephosphate	C00111	3.74E-04	0.04545455	12	0.20832062	2.6695194	-0.63324761	23.482065	0	0	44.11695	15.676527	0	-1.97	0.213	0
	PRPP	C00119	2.58E-04	0	4	0.2235039	2.9666615	-0.61574328	28.216454	0	1	47.43403	47.538738	0	-4.067	0.108	0
	fumarate	C00122	1.15E-04	0.2	6	0.48380318	2.3402433	-0.70523691	0	0	0	31.61699	0	0	-0.166	0.203	1
	glutathione disulfide	C00127	2.37E-03	0	1	0.36967212	2.8963675	-0.49042407	107.34464	0	0	209.6737	60.363499	0	-5.829	0.042	0
	inosine-monophosphate	C00130	2.72E-04	0.04761905	7	0.54043353	2.9654498	-0.55700958	8.5307722	12.949531	3	54.13358	18.004448	1	-4.173	0.112	0
	dATP	C00131	1.55E-05	0	1	0.34398293	2.9562936	-0.6279754	25.762857	0	3	61.48356	50.375816	1	-4.016	0.104	0
E.coli	histidine	C00135	6.76E-05	1	2	0.46655276	2.7470851	-0.38786784	33.978001	0	1	49.5071	0	0	-3.727	0.161	0
	myo-inositol	C00137	5.72E-06	0	4	0.54915386	3.0062153	-0.46671078	0	0	1	50.75112	0	1	-1.427	0.091	0
	GMP	C00144	2.37E-05	0.13333333	6	0.46404621	2.9655471	-0.56711	8.5307722	12.949531	3	54.13358	18.004448	1	-4.21	0.104	0
	adenine	C00147	1.47E-06	0.04761905	7	0.75696397	2.4494536	-0.44574562	0	0	2	6.699551	2.3279202	1	-0.148	0.175	0
	proline	C00148	3.85E-04	0	1	0.39176998	2.9519591	-0.41063055	34.464172	0	1	58.61605	0	0	-2.413	0.251	0
	malate	C00149	1.68E-03	0.06666667	6	0.41447622	2.6853454	-0.70714152	31.940584	0	0	24.26701	0	0	-1.52	0.156	1
	asparagine	C00152	5.11E-04	1	2	0.33203515	2.7589712	-0.45936516	8.5307722	0	0	22.50805	0	0	-3.544	0.152	0
	homocysteine	C00155	3.70E-04	0	3	0.39508834	2.809371	-0.40908334	25.762857	0	0	40.2926	0	0	-2.072	0.218	0
	4-hydroxybenzoate	C00156	5.22E-05	0	4	0.79825246	2.4755485	-0.60199398	0	0	1	73.90104	5.2434282	1	1.557	0.222	0
	citrate	C00158	1.96E-03	0.33333333	4	0.28641728	2.8216975	-0.70779455	63.881168	0	0	31.61699	0	0	-1.998	0.126	1
	phenylpyruvate	C00166	8.98E-05	0	3	0.67851609	2.6192956	-0.52533448	17.232086	0	1	97.05951	0	1	0.524	0.25	0
	UDP-glucaronate	C00167	5.66E-04	0	2	0.51576704	2.9818482	-0.48698521	91.78199	12.949531	3	117.3761	56.233585	2	-4.662	0.074	0
	valine	C00183	4.02E-03	0	1	0.2142	2.8494208	-0.31706995	29.857702	0	0	67.72499	66.652031	0	-2.286	0.231	0
	threonine	C00188	1.79E-04	0.1	5	0.36300907	2.7927809	-0.3988986	12.796158	0	0	44.56652	33.326015	0	-2.502	0.183	0
	2,3-Dihydroxybenzoic acid	C00196	1.38E-04	0	2	0.71984166	2.4962699	-0.61067373	2.2085397	0	1	66.55106	10.486856	1	1.622	0.168	0
	3-phosphoglycerate	C00197	1.54E-03	0	3	0.22090781	2.7090659	-0.68446761	8.5307722	0	0	30.51699	15.676527	0	-2.337	0.186	0
	adenosine	C00212	1.31E-07	0.16666667	4	0.5671671	2.9579263	-0.42974123	0	0	3	54.13358	2.3279202	1	-2.158	0.074	0
	adenosine-phosphosulfate	C00224	6.63E-06	0	3	0.46371472	2.9662502	-0.50736594	8.5307722	0	3	54.13358	18.513605	1	-3.886	0.114	0
	acetyl phosphate	C00227	1.07E-03	0	2	0.123	2.5667305	-0.61868048	12.796158	0	0	29.40845	49.002541	0	-1.298	0.304	0
	guanine	C00242	1.88E-04	0	4	0.7663275	2.6034517	-0.50635815	0	12.949531	2	6.699551	2.3279202	1	-1.158	0.141	0
	gluconate	C00257	4.16E-05	0	2	0.52300155	2.87363	-0.49408355	0	0	0	55.89255	0	0	-3.106	0.102	0
	glycerate	C00258	1.41E-03	0.16666667	4	0.47414967	2.648561	-0.56844461	0	0	0	30.51699	0	0	-1.942	0.186	0
	uridine	C00299	2.09E-03	0	3	0.56693256	2.9739747	-0.46144807	29.00082	12.949531	2	83.54203	23.862217	1	-2.219	0.123	0
	citrulline	C00327	1.35E-03	0	3	0.48562169	2.8458133	-0.43099335	47.485954	0	0	58.61605	23.862217	0	-3.357	0.129	0
	deoxyguanosine	C00330	5.22E-07	0	1	0.54351074	2.9483972	-0.45614555	17.232086	12.949531	3	61.48356	2.3279202	1	-2.612	0.083	0
	acetoacetyl-CoA	C00332	2.18E-05	0	3	0.35369542	2.9816422	-0.52552813	167.98883	12.949531	3	282.6371	182.57339	1	-4.125	0.059	0
	dihydroorotate	C00337	1.19E-05	0	2	0.47469416	2.8287699	-0.50979954	8.5307722	0	1	22.50805	23.862217	1	-0.751	0.156	0
	6-phospho-D-gluconate	C00345	3.77E-03	0	3	0.45794708	2.8827114	-0.67311466	8.5307722	0	0	55.89255	15.676527	0	-4.157	0.122	0
	glucosamine-6_phosphate	C00352	1.15E-03	0	4	0.46720096	2.9792151	-0.52128768	8.5307722	0	1	54.13358	15.676527	1	-3.251	0.138	0
	fructose-1,6-bisphosphate	C00354	1.52E-02	1	2	0.3571339	2.9941537	-0.67311382	32.012836	0	1	69.49251	31.353054	0	-4.186	0.107	0
	dAMP	C00360	8.84E-06	0	1	0.46939987	2.9536128	-0.59084308	25.762857	0	3	61.48356	18.004448	1	-2.695	0.126	0
	dGMP	C00362	5.07E-05	0	1	0.42896968	2.9550786	-0.566746	25.762857	12.949531	3	61.48356	18.004448	1	-3.679	0.115	0
	dTDP	C00363	3.78E-04	0	3	0.3637363	2.9720819	-0.60499483	57.51347	12.949531	2	106.7005	89.050446	1	-3.113	0.118	0
	cytosine	C00380	1.41E-05	0	2	0.62987578	2.544245	-0.4479351	16.745916	0	1	36.108	23.862217	1	-1.846	0.201	0
	guanosine	C00387	1.62E-06	0	3	0.5537346	2.9594364	-0.47204834	0	12.949531	3	54.13358	2.3279202	1	-3.142	0.073	0
	aconitate	C00417	1.61E-05	1	2	0.26025486	2.5890384	-0.70504045	31.940584	0	0	33.82553	0	0	-0.848	0.141	0
	N-acetyl-ornithine	C00437	4.33E-05	0	3	0.32066476	2.8514755	-0.40566367	48.605381	0	0	88.0245	33.326015	0	-3.697	0.151	0
	carbamyl-aspartate	C00438	5.90E-04	0	2	0.51816219	2.7567587	-0.65899742	31.940584	0	0	22.50805	23.862217	0	-1.763	0.115	0
	dCTP	C00458	3.45E-05	0.33333333	3	0.46872878	2.9685774	-0.6180737	42.508774	0	2	90.89201	71.91011	1	-4.287	0.108	0
	dTTP	C00459	4.62E-03	0	1	0.25869229	2.972435	-0.62093496	57.51347	12.949531	2	106.7005	105.23613	1	-3.773	0.107	0
	cytidine	C00475	2.59E-06	0	4	0.5534057	2.9711962	-0.44764203	16.745916	0	2	83.54203	23.862217	1	-2.195	0.093	0
	shikimate	C00493	1.41E-05	0	2	0.50890738	2.8945942	-0.53323692	17.232086	0	1	59.20109	0	1	-1.162	0.136	0
	ADP-glucose	C00498	4.27E-06	0	2	0.54069495	2.9733961	-0.47724292	48.072674	0	4	110.0261	34.699287	2	-4.122	0.074	0
	deoxyadenosine	C00559	2.82E-06	0	2	0.58064985	2.9467888	-0.39794385	17.232086	0	3	61.48356	2.3279202	1	-1.606	0.084	0
	cyclic-AMP	C00575	3.52E-05	0	1	0.55597728	3.0084202	-0.49179345	27.747452	18.713068	4	54.13358	18.513605	2	-2.373	0.129	0
	histidinol	C00860	1.28E-05	1	2	0.56515914	2.7972381	-0.32017964	33.978001	0	1	71.56558	0	0	-1.819	0.182	0
	quinolinate	C03722	1.15E-05	0	2	0.55568278	2.4681509	-0.69295162	18.954454	0	1	60.82459	0	1	-0.042	0.179	0
	ATP	C00002	3.39E-03	0.5	4	0.42522323	2.9664838	-0.62666225	8.5307722	0	3	54.13358	50.375816	1	-4.546	0.096	1
	NAD+	C00003	4.70E-04	0.10714286	8	0.54069591	2.9688232	-0.51378423	46.96413	12.949531	5	175.9922	34.699287	2	-7.801	0.063	0
	NADH	C00004	4.50E-04	0.16666667	4	0.54058594	2.9802225	-0.52770728	57.703442	0	5	175.9922	34.699287	2	-4.689	0.07	0
	ADP	C00008	6.30E-04	0.5	4	0.46371293	2.9660692	-0.63219053	8.5307722	0	3	54.13358	34.190132	1	-3.886	0.104	0
	AMP	C00020	1.60E-04	0.03157895	20	0.50219971	2.9641578	-0.5823155	8.5307722	0	3	54.13358	18.004448	1	-3.225	0.113	0
	Pyruvate	C00022	1.24E-03	0.03921569	18	0.16193631	2.4755504	-0.5895986	12.796158	0	0	29.40845	33.326015	0	-1.244	0.336	0
	L-Glutamate	C00025	8.18E-02	0.02205882	17	0.30460405	2.8003836	-0.59298134	40.471355	0	0	38.31654	0	0	-2.694	0.155	1
	2-Oxoglutarate	C00026	1.11E-03	0.02777778	9	0.19902514	2.6908374	-0.66512686	40.471355	0	0	31.61699	0	0	-1.303	0.2	0
	GDP	C00035	2.20E-04	0.33333333	3	0.42566904	2.9674129	-0.62751305	8.5307722	12.949531	3	54.13358	34.190132	1	-4.87	0.097	0
	L-Alanine	C00041	2.47E-02	0.2	5	0.2142	2.6955085	-0.37000412	12.796158	0	0	36.108	33.326015	0	-3.124	0.254	1
	Succinate	C00042	3.40E-04	0	4	0.27618599	2.6600556	-0.70726633	46.478542	0	0	31.61699	0	0	-0.526	0.192	1
	GTP	C00044	5.90E-04	0.14285714	7	0.39961782	2.9678149	-0.62380373	8.5307722	12.949531	3	54.13358	50.375816	1	-5.531	0.091	1
	L-Aspartate	C00049	1.57E-02	0	8	0.30306789	2.7412763	-0.64470696	31.940584	0	0	22.50805	0	0	-2.412	0.156	1
	L-Arginine	C00062	3.58E-02	0	3	0.30467409	2.8467581	-0.37197614	25.762857	43.095066	0	58.61605	0	0	-3.517	0.129	0
	L-Glutamine	C00064	8.04E-02	0.1	5	0.33341646	2.8105457	-0.44240996	34.464172	0	0	38.31654	0	0	-3.375	0.152	1
	Phosphoenolpyruvate	C00074	1.90E-04	0	3	0.19482203	2.5316682	-0.68372554	0	0	0	37.86697	47.777321	0	-1.006	0.24	0
	L-Tryptophan	C00078	3.00E-05	0	4	0.67442864	2.7640889	-0.40016633	42.886093	0	2	117.1582	0	1	-1.566	0.159	0
	L-Phenylalanine	C00079	3.50E-04	0	2	0.61489522	2.7547119	-0.38270196	17.232086	0	1	103.7591	0	1	-1.556	0.191	0
S.cerevisiae	L-Tyrosine	C00082	1.60E-04	0	1	0.59373242	2.7553606	-0.43409511	17.232086	0	1	96.40908	5.2434282	1	-2.223	0.162	0
	D-Fructose 6-phosphate	C00085	3.20E-04	0	5	0.49413991	2.9892137	-0.58462608	8.5307722	0	1	69.49251	15.676527	0	-3.127	0.135	0
	D-Glucose 6-phosphate	C00092	3.70E-03	0	4	0.4905296	2.9625309	-0.58356768	8.5307722	0	1	55.89255	15.676527	1	-3.281	0.135	1
	sn-Glycerol 3-phosphate	C00093	5.00E-04	0	3	0.3567448	2.7578208	-0.6300028	8.5307722	0	0	52.57547	15.676527	0	-2.59	0.208	0
	D-Glucose 1-phosphate	C00103	9.00E-05	0	4	0.50497961	2.9663177	-0.55763352	39.541901	0	1	55.89255	15.676527	1	-2.677	0.136	0
	Glycerone phosphate	C00111	7.20E-04	0.13333333	6	0.20832062	2.6695194	-0.63324761	23.482065	0	0	44.11695	15.676527	0	-1.97	0.213	0
	D-Ribose 5-phosphate	C00117	4.10E-04	0	6	0.49309793	2.9520912	-0.629421	8.5307722	0	1	47.43403	15.676527	0	-3.131	0.154	0
	Fumarate	C00122	1.65E-03	0	4	0.48380318	2.3402433	-0.70523691	0	0	0	31.61699	0	0	-0.166	0.203	1
	GMP	C00144	6.00E-05	0.2	5	0.46404621	2.9655471	-0.56711	8.5307722	12.949531	3	54.13358	18.004448	1	-4.21	0.104	0
	Adenine	C00147	5.00E-05	0	4	0.75696397	2.4494536	-0.44574562	0	0	2	6.699551	2.3279202	1	-0.148	0.175	0
	Malate	C00149	1.68E-03	0.06666667	6	0.41447622	2.6853454	-0.70714152	31.940584	0	0	24.26701	0	0	-1.52	0.156	1

	L-Asparagine	C00152	4.66E-03	0	2	0.33203515	2.7589712	-0.45936516	8.5307722	0	0	22.50805	0	0	-3.544	0.152	0
	Citrate	C00158	1.47E-03	0.33333333	3	0.28641728	2.8216975	-0.70779455	63.881168	0	0	31.61699	0	0	-1.998	0.126	1
	3-Phospho-D-glycerate	C00197	8.90E-04	0	3	0.22090781	2.7090659	-0.68446761	8.5307722	0	0	30.51699	15.676527	0	-2.337	0.186	0
	D-Ribulose 5-phosphate	C00199	2.00E-05	0	5	0.42681855	2.8199623	-0.57482624	8.5307722	0	0	61.03399	15.676527	0	-3.058	0.152	0
	Isocitrate	C00311	1.90E-04	0.0952381	7	0.4389635	2.8126755	-0.70789599	42.213158	0	0	26.47555	0	0	-2.072	0.112	0
	6-Phospho-D-gluconate	C00345	8.70E-04	0	2	0.45794708	2.8827114	-0.67311466	8.5307722	0	0	55.89255	15.676527	0	-4.157	0.122	0
	D-Fructose 1,6-bisphosphate	C00354	8.76E-03	1	2	0.3571339	2.9941537	-0.67311382	32.012836	0	1	69.49251	31.353054	0	-4.186	0.107	0
	Pantothenate	C00864	2.10E-04	0	2	0.34613365	2.923965	-0.43633202	79.255997	12.949531	0	125.4419	66.652031	0	-1.494	0.115	0
	L-Serine	C00065	0.00077	0	6	0.39069223	2.7133796	-0.44136977	0	0	0	28.75803	0	0	-2.811	0.187	0
	D-Glucose 1-phosphate	C00103	0.00023	0	7	0.50497961	2.9663177	-0.55763352	39.541901	0	1	55.89255	15.676527	1	-2.677	0.136	0
	D-Ribose 5-phosphate	C00117	0.00024	0	5	0.49309793	2.9520912	-0.629421	8.5307722	0	1	47.43403	15.676527	0	-3.131	0.154	0
	D-Glyceraldehyde 3-phosphate	C00118	0.00012	0.04444444	10	0.2391061	2.7103465	-0.63274568	8.5307722	0	0	30.51699	15.676527	0	-2.131	0.213	0
	L-Leucine	C00123	0.00123	0	1	0.2142	2.8748624	-0.33988363	47.089787	0	0	83.53349	66.652031	0	-1.667	0.22	1
Bacillus subtilis	L-Valine	C00183	0.00601	0	1	0.2142	2.8494208	-0.31706995	29.857702	0	0	67.72499	66.652031	0	-2.286	0.231	0
	(S)-Lactate	C00186	0.00369	0	1	0.31986156	2.6253042	-0.57461077	12.796158	0	0	37.86697	33.326015	0	-0.734	0.248	0
	L-Threonine	C00188	0.00081	0	3	0.36300907	2.7927809	-0.3988986	12.796158	0	0	44.56652	33.326015	0	-2.502	0.183	0
	D-Ribulose 5-phosphate	C00199	0.00043	0	5	0.42681855	2.8199623	-0.57482624	8.5307722	0	0	61.03399	15.676527	0	-3.058	0.152	0
	Isocitrate	C00311	0.00026	0.2	5	0.4389635	2.8126755	-0.70789599	42.213158	0	0	26.47555	0	0	-2.072	0.112	0
	L-Isoleucine	C00407	0.00037	0	1	0.2142	2.8866551	-0.31279653	38.388474	0	0	83.53349	66.652031	0	-1.757	0.22	0

Figure S1. Distribution of predictive errors in both training set (a) and test set (b).

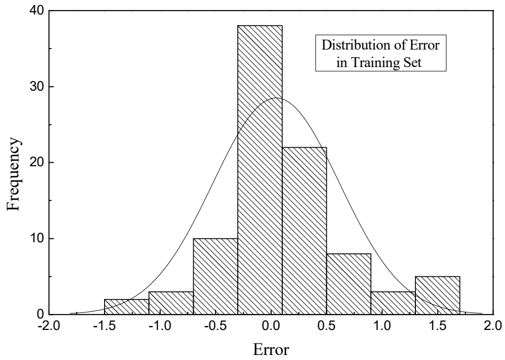

(a)

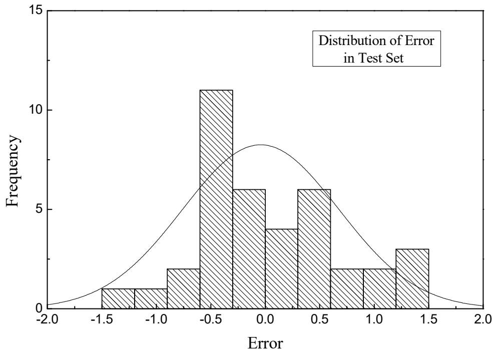

(b)
